# Functional health state description and valuation by people aged 65 and over: a pilot study

**DOI:** 10.1186/s12877-018-0711-9

**Published:** 2018-01-16

**Authors:** Riaan Botes, Karin M. Vermeulen, Adelita V. Ranchor, Erik Buskens

**Affiliations:** 1 Department of Epidemiology, University Medical Center Groningen, PO Box 30.001, 9700 RB Groningen, The Netherlands; 2 Department of Health Psychology, University of Groningen, University Medical Center Groningen, PO Box 196, 9700 AD Groningen, The Netherlands

**Keywords:** Elderly, Quality of life, Functioning, Health states

## Abstract

**Background:**

Assessing quality of life among the elderly is a complex and multifaceted issue. Elderly people might find valuing and describing their personal experience of quality of life (QoL) demanding and cumbersome. This study therefore sought to determine the feasibility of administering two questionnaires in two samples of elderly people.

**Methods:**

A preference-based instrument (EQ-5D + C) and a currently achieved functioning questionnaire (CAF) were utilized. Two pilot studies were performed. The first was performed in South Africa (*n* = 30), designed to test whether elderly respondents could complete and understand the two questionnaires and also to indicate which valuation method, visual analogue scale or time trade off they preferred. A second pilot study was performed in the Netherlands (*n* = 30), designed to investigate the use of both questionnaires in determining quality of life and health state valuations in a Dutch sample of elderly.

**Results:**

Seventy percent of the South African respondents indicated that they preferred the visual analogue scale (VAS) method, when compared to the time trade-off (TTO). In both the South African and the Dutch pilot studies, the respondents, with different dependency levels, were able to use both questionnaires to determine health state descriptions and valuations. When ranking the profiles from fewer to more problems, the EQ-5D + C exhibits a gradual downwards trend, with a maximum of 100 and minimum VAS value of 41. The CAF also exhibits a gradual downwards trend, with a maximum of 1.00 and minimum VAS value of 36.

**Conclusions:**

The results indicate that individuals from different parts of the world are able to complete, describe, and value the questionnaires. It is our recommendation that a comprehensive study should be done, which includes both the EQ-5D + C questionnaire and the CAF questionnaire, since the two questionnaires have proven to be feasible in providing information on quality of life and well-being of elderly people.

**Electronic supplementary material:**

The online version of this article (10.1186/s12877-018-0711-9) contains supplementary material, which is available to authorized users.

## Background

Aging is a progressive process of deterioration, comprising physical and mental domains. It constitutes loss of ability to maintain and function at previous levels of achievement. Moreover, aging is a personal process that includes both positive and negative experiences [1]. Quality of life (QoL) is one popular outcome measurement used in assessing the effects of aging on what people judge to be important in their lives. It is, however, a contentious subject, since some are of the opinion that i) the elderly population should generate the health descriptions and valuations relating to QoL, while others consider that ii) the general population should provide these health descriptions and valuations [2–4]. Furthermore, the methods and procedures used to determine QoL have yet to be standardized, thus raising issues of the accuracy and applicability of elderly health state descriptions and valuations [5, 6].

A common method is to use a generic classification system such as the EQ-5D to value and describe health status [7]. Typically, the EQ-5D describes health status in terms of health-related quality of life (HRQoL) domains: mobility, self-care, usual activities, pain/discomfort, and anxiety/depression [8]. Despite its many advantages, some concerns have been raised with regard to its use among the elderly. One of the concerns that has been raised is that it may be insufficiently responsive to elements of quality of life and well-being [9]. Applying a totally different conceptual framework such as the capability approach [10] might prove to be a very effective way of defining actual disability, as well as for investigating the valuation of functioning as disability continues [11].

An instrument like the EQ-5D incorporates HRQoL to a large extent into the valuation and descriptive capacity, while the capability approach incorporates non-health aspects such as attachments, role, security, control, and enjoyment, which will influence health and, ultimately, overall well-being [12, 13].

It has been suggested that the notion of disability and loss of functioning might specifically apply to the elderly as this process is to some degree linked to chronological age and tends to evolve slowly [14]. The functioning of the elderly might largely depend on their acquired lifetime personal, social, and financial assets [14, 15].

Previous studies have shown that a generic preference-based questionnaire, like the EQ-5D, and a functioning questionnaire provide complimentary information on quality of life [16]. It is, however, still uncertain which domains and dimensions of the questionnaires overlap and to what extent the domains would be double-counted using the two instruments [17].

The present pilot study aimed to determine the feasibility of two distinct components. First, our aim was to determine which of the two methods for elucidating health-state valuations would be appropriate for the elderly, that is, whether to use a visual analogue scale (VAS) or apply a Time Trade Off method (TTO).

Second, we wished to study the feasibility of administering the EQ-5D + C and a generally accepted functioning questionnaire (CAF) to elderly people both from South Africa and the Netherlands.

## Methods

A pilot study was performed with two different samples. The first sample was recruited among South African home-dwelling elderly, 65 years and older. The South African elderly participants were asked to complete health-state descriptions and valuations in a recently developed Currently Achieved Functioning questionnaire (CAF) and the EQ5D + C [18] in order to determine the feasibility of using these questionnaires in an elderly population.

The results of the South African study were reflected upon, and subsequently a second pilot study was performed in the Netherlands. The two questionnaires were evaluated and compared in terms of their suitability for establishing appropriate health state descriptions and valuations for elderly subgroups. The purpose of the inclusion of these questionnaires was to extract descriptive information regarding the individual domains of the EQ-5D + C and the CAF.

The interviews were performed in accordance with the Helsinki declaration. Ethical approval was granted by the medical ethical testing committee of the University Medical Center Groningen, ethics number M11.098466 (Additional file 1).

The UMCG ethical testing committee found that according to medical ethical law the pilot study is not regarded as a study involving significant intervention in human beings. The pilot study is also part of a PhD project, the major part of which will be performed in the Netherlands; therefore, no additional ethical approval was sought in South Africa.

Written consent was however obtained from each respondent who agreed to take part in the study.

### Questionnaires

#### Currently achieved functioning (CAF) questionnaire

The Currently Achieved Functioning questionnaire was developed to investigate the achieved functioning and not the functional aspirations or capabilities of the elderly respondents. The CAF questionnaire included the attachment, enjoyment, security, role, and control attributes, with five response categories possible. Inspiration for the development of the CAF came from the work performed by Grewal and colleagues [19]. They embarked on a 2-stage analysis, first, to determine factors that contribute to the quality of elderly informants’ lives and, second, to identify the attributes of quality of life. From this study, 5 attributes emerged: *Attachment, Enjoyment, Security, Role, and Control.* Additional file 2 summarizes the aspects that contribute and determine these attributes.

Coast and colleagues investigated this matter further by doing qualitative and quantitative work on these five attributes [20]. The qualitative work focused on the design of a measurement instrument, while the quantitative work focused on the validation of the measurement instrument. Ultimately, an instrument to determine the effect of health and social care interventions was presented, while mentioning the potential of the instrument in the economic evaluation of interventions [20].

The work performed by Grewal and colleagues to determine qualitative attributes important to the elderly was utilized, since these attributes fit into the theory of the capability approach [19]. An extract from the research done by the authors summarizes what was done:

“This paper reports an attempt to determine attributes for a new index clearly focusing on quality of life for older people rather than health or other influences on quality of life. In-depth interviews were conducted with 40 purposively selected informants aged 65 and over in private households to explore their views about what is important to them in terms of quality of life. Data were analyzed using Framework qualitative analysis. Initial discussions tended to concentrate upon factors influencing quality of life including activities, relationships, health, wealth and surroundings. Further probing and analysis suggested five conceptual attributes: attachment, role, enjoyment, security and control.”

Subsequent literature suggests that the non-health-related attributes of attachment – enjoyment, security, role, and control – are unique and can possibly be an alternative or at least contribute to current healthcare interventions designed for the elderly [21].

The validity of a questionnaire, which includes the attachment, enjoyment, security, role, and control attributes, was also tested in a Dutch setting, with positive results [13].

The original version of the CAF questionnaire was constructed in English (Additional file 3), to be utilized in South Africa, while the English version was translated into Dutch by a specialist translator and one of the authors of the paper (AR).

#### EQ-5D + C questionnaire

We used an extended version (EQ-5D + C) of the standard EQ-5D that included “cognitive functioning” as an additional attribute [22]. The standard EQ-5D classification system developed by the EuroQol Research Foundation (https://euroqol.org/) describes health status according to five attributes: mobility, self-care, usual activities, pain/discomfort, and anxiety/depression. Each attribute has three levels: “no problems” (“1”), “some problems” (“2”), and “severe problems” (“3”). Health state descriptions are constructed by choosing one level for each attribute (e.g., the best health state is represented by 11,111).

The non-standard EQ-5D + C is similar to the EQ-5D, but with a 3-level cognition attribute added, Additional file 4. Of specific relevance to the elderly are health aspects such as vision and hearing, and in particular cognition [23–25]. The addition of the cognition domain makes the EQ-5D + C of specific importance to the elderly, since aging is to a degree associated with a decline in cognitive ability.

### Participants

#### South African sample

Thirty independent-living elderly individuals from the general population were recruited and divided into to three groups, ten individuals in each group. To be included in the study, participants had to be South African citizens, 65 years and older, and living independently in the Bloemfontein area. Participants were recruited through referrals from elderly community leaders. Potential participants were contacted by telephone and asked if they would be willing to participate. Elderly people willing to participate were recruited, and one-on-one interviews were conducted by a trained interviewer.

#### Dutch sample

After reflecting on the results of the first pilot study, the decision was made to perform a second pilot study in the Netherlands. The CAF and the EQ5D + C were included in the Dutch pilot study. Thirty elderly were recruited in the Groningen area. Ten were living independently, another ten individuals were semi-independent, meaning they lived independently in the neighborhood of an elderly care center, from which they could receive some help with regard to household chores, dinner, and medical care, and the last group were living in an elderly care center.

### Procedure

#### South African sample

The first group was presented with a questionnaire and was asked to value applicable EQ5D + C target health states, using a Time Trade Off (TTO), Additional file 5 and a visual analogue scale (VAS), Additional file 6.

Previous studies have shown that TTO techniques place a great cognitive burden on respondents, since they require a high degree of abstract reasoning [26]. Taking this into consideration, the decision was made to utilize a simplified version of the TTO exercise [27].

In our pilot and feasibility study we therefore switched to a more simplistic (more crude steps) analogue version of the TTO method. The TTO technique required the respondents to value how much time in health state 111111 (full health) was equivalent to 10 years spent in a target state. Target states represent different levels of decline in HRQoL. Thus, a typical TTO valuation task would involve a hypothetical trade-off between length and quality of life. The TTO process, utilized in our study, provides the elderly respondents with options to choose from, rather than subjectively reasoning and cognitively determining the point of indifference. The chosen TTO exercise provided elderly respondents with a less cognitively burdensome alternative.

The target states were 112112, 212111, 111221, 212121, 133113, 212321, 333211, 323331, and 333333. Only 9 health states were valued for the TTO exercise, since health state 111,111 was given as the comparison full-health state.

With regard to the valuation method, we were mainly interested in the feasibility of TTO as a measurement tool in a specific frail elderly population. In line with previous studies our simplified TTO again proved to be too complicated for the majority of respondents, and we subsequently omitted it from the Dutch sample.

The VAS method requires ten health states, 111111, 112112, 212111, 111221, 212121, 133113, 212321, 333211, 323331, and 333333, rated on a visual analogue scale, typically ranging from 0 (worst off) to 100 (full health). They were asked to state which of the two techniques was the easiest to complete in terms of understanding the task that had to be completed, and also the cognitive burden of the task. Upon investigating the results from the first group, it was decided to continue only with the VAS valuations in group two and group three.

The second group was asked to value and describe ten EQ-5D + C health states using a visual analogue scale. The health states chosen were 111111, 112112, 212111, 111221, 212121, 133113, 212321, 333211, 323331, and 333333.

The third group was asked to complete the questionnaire pertaining to functioning (CAF) that they were currently achieving and also to value ten health states using a VAS: 11111, 21114, 12335, 55555, 11245, 44433, 11122, 11312, 33333, and 33544.

Care was taken to instruct the individuals not to consider their own health when valuing the health states. Instead they had to view the valuation procedure as a task regarding a hypothetical state. It was also made clear that the value of 100 on the VAS (visual analogue scale) would be considered to be the best possible value attainable and that the 0 value would be considered equal to death. The respondents were also instructed to consider the whole scale and not just the marked intervals [28].

The health states were chosen randomly to reflect the better and worse-off states associated across the spectrum of the two questionnaires. Care was taken, however, to include the health states that represented full health and worst possible health. Only ten health states per questionnaire were included, so as not to impose a heavy cognitive burden on the elderly respondents [26].

#### Dutch sample

The elderly respondents completed the CAF and EQ-5D + C questionnaires and again three subgroups, dependent, semi-dependent, and independent respondents, completed 10 hypothetical health state valuations, for each questionnaire using a VAS.

The health states were identical to the health states that were valued in the South African pilot study. Health states 111111, 112112, 212111, 111221, 212121, 133113, 212321, 333211, 323331, and 333333 for the EQ-5D + C, while health states 11111, 21114, 12335, 55555, 11245, 44433, 11122, 11312, 33333, and 33544 were valued for the CAF questionnaire.

## Results

The demographic information on all four groups is presented in Table 1. Seventy percent of the respondents from the SA sample indicated that they preferred the VAS method as compared to the TTO. Exemplary comments from the respondents with regard to the task were:

**Table 1:** Demographic data for all *respondents*

SA pilot study			
Demographic factor	Group 1	Group 2	Group 3
	*N* = 10	*N* = 10	*N* = 10
Male (%)	60	60	40
Age (mean)	71.4	68.6	68
Marital Status			
Single (n)	1	0	0
Married (n)	6	8	9
Widowed (n)	2	2	1
Divorced (n)	0	0	0
Education			
High school (n)	5	1	2
Diploma (n)	1	3	0
Degree (n)	4	5	6
Post degree (n)	0	1	2
Chronic diseases (number)			
1	2	4	5
2	6	5	5
3	3	1	0
Dutch pilot study			
	Independent living	Semi-dependent	Care center
	*N* = 10	*N* = 10	*N* = 10
Age (mean)	73,5	86,4	83,2
Male (%)	40%	20%	20%
Education			
- Primary school	0	5	6
- High School	1	2	1
- Diploma	3	2	2
- University	6	1	1
Percentage of people living alone	40%	80%	90%
Religious background (%)	60%	70%	50%
Total number of chronic disease	11	32	25

“The TTO exercise placed a heavy cognitive burden on me”; “I feel the TTO exercise is too difficult to complete”; “the VAS is much easier to complete”; and “I feel the TTO exercise might not provide accurate results.” Based on the fact that respondents complained about and failed to complete the TTO exercise, it was decided to continue using the VAS in group two and group three in the South African study, and in all groups in the Dutch study.

Ranking the valuations of the South African EQ-5D + C health states from best to worst health state, illustrated in Table 2, the EQ-5D + C exhibits a gradual downwards trend, with a maximum of 100 and minimum VAS value of 41. The achieved functioning questionnaire also exhibits a gradual downwards trend, with a maximum of 1.00 and a minimum VAS value of 36 (Table 3).

**Table 2:** EQ-5D + C questionnaire ranked health states according to average VAS values

	Dutch Independent	Dutch Semi-dependent	Dutch Dependent	SA Independent
111111	86	78	82	100
112112	78	72	73	81
212111	75	69	74	79
111221	72	69	72	78
212121	72	65	72	77
133113	62	53	68	60
212321	60	62	66	58
333211	55	43	64	56
323331	44	38	63	52
333333	35	37	60	41

**Table 3:** CAF questionnaire ranked health states according to average VAS values

	Dutch Independent	Dutch Semi-dependent	Dutch Dependent	SA Independent
11111	85	80	77	100
11,122	84	74	78	99
11245	65	58	66	90
11312	83	78	75	84
12335	70	58	68	69
21114	78	73	76	67
33333	73	67	68	67
33544	58	57	67	42
44433	58	54	60	41
55555	47	48	57	36

Table 4 summarizes the results from the EQ-5D + C health state descriptions. The South African and Dutch elderly had no difficulty with completing the health state descriptions. The CAF questionnaire also performed adequately, with evident discriminatory power between the functioning dimensions of the questionnaire, Table 5.

**Table 4:** EQ-5D + C description results

South African pilot study			
*n* = 10	No.	Some	Extreme
Independent living			
Mobility	7	3	0
Self-care	9	1	0
Usual activities	6	4	0
Pain/Discomfort	5	3	2
Anxiety/Depression	9	1	0
Cognition	7	3	0
Dutch pilot study			
Independent living			
*n* = 10			
Mobility	5	5	0
Self-care	10	0	0
Usual activities	8	2	0
Pain/Discomfort	8	2	0
Anxiety/Depression	9	1	0
Cognition	9	1	0
Semi-dependent			
*n* = 10			
Mobility	0	10	0
Self-care	8	0	2
Usual activities	2	6	2
Pain/Discomfort	2	4	4
Anxiety/Depression	9	0	1
Cognition	6	4	0
Care center			
*n* = 10			
Mobility	0	8	2
Self-care	6	2	2
Usual activities	6	1	3
Pain/Discomfort	5	3	2
Anxiety/Depression	9	0	1
Cognition	9	1	0

**Table 5:** Functioning description results

	South African				
	I have all	I have a lot	I have some	I have a little	I have none
Attachment	3	5	1	1	0
Enjoyment	2	5	3	0	0
Security	1	4	4	1	0
Role	2	4	3	1	0
Control	5	3	1	1	0
Dutch independent living					
Attachment	5	3	2	0	0
Enjoyment	3	6	1	0	0
Security	1	3	5	1	0
Role	4	6	0	0	0
Control	5	4	1	0	0
Dutch semi-independent					
Attachment	4	2	3	1	0
Enjoyment	2	2	5	1	0
Security	1	5	2	1	0
Role	3	3	2	2	0
Control	4	2	1	0	3
Dutch dependent					
Attachment	1	3	3	3	0
Enjoyment	2	2	5	1	0
Security	2	2	5	0	1
Role	2	5	2	1	0
Control	4	3	3	0	0

The results of the EQ5D + C subgroup valuations can be seen in Table 2. The EQ-5D + C for the Dutch independent group exhibits a gradual downwards trend, with a maximum of 86 and minimum VAS value of 35. The Dutch semi-dependent group exhibits a gradual downwards trend, with the exception of health state 212321. A maximum of 78 and minimum VAS value of 37 were found. The Dutch dependent group also exhibits a gradual downward trend, with a maximum of 82 and minimum VAS value of 60 found.

As for the subgroups of health state valuations of the CAF questionnaire, the Dutch independent group had a maximum of 85 and a minimum VAS value of 47. The Dutch semi-dependent exhibited an 80 maximum and minimum VAS value of 48. The Dutch dependent group exhibited a maximum of 77 and minimum VAS value of 57.

## Discussion

The aim of this study was to investigate which of the two methods, TTO or VAS, which elucidate health state valuations from the elderly, would be appropriate. Also to test the feasibility of using the EQ-5D + C and the CAF questionnaires, in two samples of elderly, in order to report on their own health and QoL.

Participants in the South African part of the pilot study remarked that they preferred the VAS to the TTO method, due to the fact that the TTO technique was cognitively more burdensome when compared to the VAS technique.

Furthermore, even with the addition of the cognition domain to the EQ-5D questionnaire, the elderly performed the health state descriptions and valuations with ease, using both questionnaires. Both the EQ-5D + C and CAF were able to extract relevant health descriptions and health state valuations. Importantly, the results of the South African study indicate that health state valuations of the EQ-5D + C and CAF questionnaire showed similar patterns. Better health states were valued closer to one, while worse off health states were valued to be closer to zero.

We decided to include both the EQ-5D + C and CAF questionnaires in the Dutch pilot study, since South African elderly were able to complete both questionnaires with ease and since both questionnaires were able to determine health descriptions and health state valuations of the different subgroups.

Valuations of health states for the EQ-5D + C and functioning questionnaire varied between the subgroups of Dutch elderly, and the health state valuation for the two questionnaires returned a high to low pattern. The EQ5D + C and the CAF questionnaire showed very little inconsistency in terms of logical order of the health state valuations. Better health states were valued closer to one, while worse off health states were valued to be closer to zero.

Previous studies have shown that capability-based questionnaires can be used to generate and value health states [29]. However the results of previous studies still indicate that it would be pertinent to still include a generic preference-based questionnaire, since the degree of double counting and missed health effects is still unclear [17].

Although controversial, the potential exists, however, to use non-health-related questionnaires to measure broader outcomes, while being more sensitive to the effects of the interventions [30]. The results of this study add to the existing literature by indicating that elderly individuals from different parts of the world and with different dependency levels are able to complete, describe, and value the health or capability states included in the questionnaires.

## Implications

It is our recommendation that a comprehensive study should be conducted, which includes both the EQ5D + C questionnaire and the CAF questionnaire, since the questionnaires would capture data on individual HRQoL and well-being dimensions. The EQ-5D + C is a version of the well-known and validated EQ-5D questionnaire but, with the inclusion of the CAF questionnaire, a study would provide a more comprehensive measure of elderly quality of life. The questionnaires could identify the subgroups of elderly at risk of diminished HRQoL and well-being. In addition, inclusion of both questionnaires would make it possible to identify domains and attributes that should be addressed in order to maximize elderly QoL and well-being.

Furthermore, such an approach might reveal relevant health state descriptions and valuations, and facilitate the planning of interventions for the elderly population.

## Conclusions

The results of the study support the feasibility of the method used in this study. This signals the necessity for a larger study to generate health state valuations and descriptions from the elderly. Valid assessment by the elderly themselves, in the domain of elderly care, QoL, and well-being, should be studied further.

## Additional files

Additional file 1: Ethical review of board. Proof of the ethical review by the Dutch Ethical board. (PDF 146 kb)

Additional file 2: Description of the 5 attributes as proposed by Grewal et al. Description of the 5 wellbeing attributes as proposed by Grewal et al. (DOCX 16 kb)

Additional file 3: CAF Questionnaire. The CAF questionnaire utilized in the study. (DOCX 12 kb)

Additional file 4: EQ5D + C questionnaire. EQ5D + C questionnaire utilized in the study. (DOCX 12 kb)

Additional file 5: TTO example. Example of the Time Trade Off method utilized in the study. (DOCX 11 kb)

Additional file 6: VAS example. Description of data: Example of the Visual Analogue Scale utilized in the study. (DOCX 13 kb)

## Abbreviations

CAF: Currently Achieved Functioning questionnaire
HRQoL: Health related quality of life
ICECAP-O: ICEpop CAPability measurement for Older people
QALY: Quality adjusted life year
QoL: Quality of life
TTO: Time trade off
VAS: Visual analogue scale

## References

[1] Steverink N, Westerhof GJ, Bode C, Dittmann-Kohli F (2001). The personal experience of aging, individual resources, and subjective well-being. J Gerontol B Psychol Sci Soc Sci.

[2] Dolan P (2000). Effect of age on health state valuations. J Health Serv Res Policy.

[3] Ebrahim S, Brittis S, Wu A (1991). The valuation of states of ill-health: the impact of age and disability. Age Ageing.

[4] Gudex C, Dolan P, Kind P, Williams A (1996). Health state valuations from the general public using the visual analogue scale. Qual Life Res.

[5] Craig BM, Busschbach JJ, Salomon JA (2009). Modeling ranking, time trade-off, and visual analog scale values for EQ-5D health states: a review and comparison of methods. Med Care.

[6] Lancsar E, Louviere J, Flynn T (2007). Several methods to investigate relative attribute impact in stated preference experiments. Soc Sci Med.

[7] Bernert S, Fernandez A, Haro JM, Konig HH, Alonso J, Vilagut G (2009). Comparison of different valuation methods for population health status measured by the EQ-5D in three European countries. Value Health.

[8] Lamers LM, McDonnell J, Stalmeier PF, Krabbe PF, Busschbach JJ (2006). The Dutch tariff: results and arguments for an effective design for national EQ-5D valuation studies. Health Econ.

[9] Holland R, Smith RD, Harvey I, Swift L, Lenaghan E (2004). Assessing quality of life in the elderly: a direct comparison of the EQ-5D and AQoL. Health Econ.

[10] Verkerk MA, Busschbach JJ, Karssing ED (2001). Health-related quality of life research and the capability approach of Amartya Sen. Qual Life Res.

[11] Mitra S (2006). The capability approach and disability. J Disabil Policy Stud.

[12] Anand P (2005). Capabilities and health. J Med Ethics.

[13] Makai P, Koopmanschap MA, Brouwer WB, Nieboer AA (2013). A validation of the ICECAP-O in a population of post-hospitalized older people in the Netherlands. Health Qual Life Outcomes.

[14] Lloyd-Sherlock P (2002). Nussbaum, capabilities and older people. J Int Dev.

[15] Kuh D (2007). A life course approach to healthy aging, frailty, and capability. J Gerontol A Biol Sci Med Sci.

[16] Davis JC, Liu-Ambrose T, Richardson CG, Bryan S. A comparison of the ICECAP-O with EQ-5D in a falls prevention clinical setting: are they complements or substitutes? Qual Life Res. 2013;22:969-77. 10.1007/s11136-012-0225-4 PMC3672090 22723152

[17] Makai P, Brouwer WB, Koopmanschap MA, Stolk EA, Nieboer AP (2014). Quality of life instruments for economic evaluations in health and social care for older people: a systematic review. Soc Sci Med.

[18] Hoeymans N, van Lindert H, Westert GP (2005). The health status of the Dutch population as assessed by the EQ-6D. Qual Life Res.

[19] Grewal I, Lewis J, Flynn T, Brown J, Bond J, Coast J (2006). Developing attributes for a generic quality of life measure for older people: preferences or capabilities?. Soc Sci Med.

[20] Coast J, Flynn TN, Natarajan L, Sproston K, Lewis J, Louviere JJ (2008). Valuing the ICECAP capability index for older people. Soc Sci Med.

[21] Couzner L, Ratcliffe J, Lester L, Flynn T, Crotty M (2013). Measuring and valuing quality of life for public health research: application of the ICECAP-O capability index in the Australian general population. Int J Public Health.

[22] Krabbe PF, Stouthard ME, Essink-Bot ML, Bonsel GJ (1999). The effect of adding a cognitive dimension to the EuroQol multiattribute health-status classification system. J Clin Epidemiol.

[23] Bryan S, Hardyman W, Bentham P, Buckley A, Laight A (2005). Proxy completion of EQ-5D in patients with dementia. Qual Life Res.

[24] Jonsson L, Andreasen N, Kilander L, Soininen H, Waldemar G, Nygaard H (2006). Patient- and proxy-reported utility in Alzheimer disease using the EuroQoL. Alzheimer Dis Assoc Disord.

[25] Selai C (2001). Assessing quality of life in dementia. Med Care.

[26] Stamuli E (2011). Health outcomes in economic evaluation: who should value health?. Br Med Bull.

[27] Gudex C. Time trade-off user manual: props and self-completion methods. na; 1994.

[28] Brazier J, Ratcliffe J, Salomon J, Tsuchiya A (2007). Measuring and valuing health benefits for economic evaluation.

[29] Couzner L, Ratcliffe J, Crotty M (2012). The relationship between quality of life, health and care transition: an empirical comparison in an older post-acute population. Health Qual Life Outcomes.

[30] Makai P, Looman W, Adang E, Melis R, Stolk E, Fabbricotti I. Cost-effectiveness of integrated care in frail elderly using the ICECAP-O and EQ-5D: does choice of instrument matter? Eur J Health Econ. 2015;16:437–50. 10.1007/s10198-014-0583-7 24760405

