# Supplementary material for: Functional health state description and valuation by people aged 65 and over: a pilot study
Source: BMC Geriatr. 2018 Jan 16;18:11. doi: 10.1186/s12877-018-0711-9 (PMC5769375; doi:10.1186/s12877-018-0711-9)
Supplement: Supplementary file 1 — Ethical review of board. Proof of the ethical review by the Dutch Ethical board. (PDF 146 kb) [file 12877_2018_711_MOESM1_ESM.pdf]

Hanzeplein 1 Postbus 30 001, 9700 RB Groningen

Universitair Medisch Centrum Groningen

Medisch Ethische Toetsingscommissie

Telefoon (050) 361 4204
Fax (050) 361 4351

Aan
Dr. K.M. Vermeulen
Epidemiologie **FA 40**

Bijlage(n)
Kenmerk M11.098466

Datum 4 maart 2011
Onderwerp METc 2011/041
Titel **Waardering van gezondheidstoestanden en kwaliteit van leven onder senioren: een pilot studie.**

De Medisch Ethische Toetsingscommissie van het Universitair Medisch Centrum Groningen (METc UMCG) heeft het bovengenoemde onderzoek besproken in haar vergadering van 3 maart 2011.

De METc UMCG is van mening dat bovengenoemd onderzoek geen medisch wetenschappelijk onderzoek met mensen is, zoals bedoeld in de Wet medisch-wetenschappelijk onderzoek met mensen (WMO).
WMO.

De METc UMCG besluit zodoende dat u geen positief WMO-oordeel behoeft, alvorens u met bovengenoemd onderzoek mag aanvangen.

Met vriendelijke groet,
namens de Medisch Ethische Toetsingscommissie,

b/a

~~prof. dr. W.A. Kamps~~
voorzitter

drs. J. Davids
ambtelijk secretaris

umcg
