# Supplementary material for: Functional health state description and valuation by people aged 65 and over: a pilot study
Source: BMC Geriatr. 2018 Jan 16;18:11. doi: 10.1186/s12877-018-0711-9 (PMC5769375; doi:10.1186/s12877-018-0711-9)
Supplement: Supplementary file 2 — Description of the 5 attributes as proposed by Grewal et al. Description of the 5 wellbeing attributes as proposed by Grewal et al. (DOCX 16 kb) [file 12877_2018_711_MOESM2_ESM.docx]

Appendix B: Description of the 5 attributes as proposed by Grewal *et al.*

| *Attachment* | Feelings of love, affection, companionship and friendship from your partner, family, friends and pets |
| --- | --- |
| *Enjoyment* | Participation in personal and group activities that is a source of pleasure and joy |
| *Security* | Feeling safe and secure, not feeling helpless when you consider factors like your finances and your health |
| *Role* | Having a purpose that provides you with a sense of value |
| *Control* | You feel independent and you make your own decisions |
