# Supplementary material for: Functional health state description and valuation by people aged 65 and over: a pilot study
Source: BMC Geriatr. 2018 Jan 16;18:11. doi: 10.1186/s12877-018-0711-9 (PMC5769375; doi:10.1186/s12877-018-0711-9)
Supplement: Supplementary file 3 — CAF Questionnaire. The CAF questionnaire utilized in the study. (DOCX 12 kb) [file 12877_2018_711_MOESM3_ESM.docx]

**Appendix C:**

Choose one option that best describes you.

***a) Attachment:***

**Feelings of love, affection, companionship and friendship from your partner, family, friends and pets**

I have all the affection, love and companionship that I want ⁯

I have a lot of the affection, love and companionship that I want ⁯

I have some of the affection, love and companionship that I want ⁯

I have a little of the affection, love and companionship that I want ⁯

I have none of the affection, love and companionship that I want ⁯

***b) Enjoyment:***

**Participation in personal and group activities that is a source of pleasure and joy**

I have all the pleasure and enjoyment that I want ⁯

I have a lot of the pleasure and enjoyment that I want ⁯

I have some of the pleasure and enjoyment that I want ⁯

I have a little of the pleasure and enjoyment that I want ⁯

I have none of the pleasure and enjoyment that I want ⁯

***c) Security:***

**Feeling safe and secure, not feeling helpless when you considering factors like your finances and your health**

I feel very safe and secure when I think about the future ⁯

I feel safe and secure when I think about the future ⁯

I feel fairly safe and secure when I think about the future ⁯

I feel unsafe and insecure when I think about the future ⁯

I feel very unsafe and very insecure about the future ⁯

***d) Role:***

**Having a purpose that provides you with a sense of value**

I do all the things that provides me with a sense of purpose ⁯

I do a lot of the things that provides me with a sense of purpose ⁯

I do some of the things that provides me with a sense of purpose ⁯

I do a little of the things that provides me with a sense of purpose ⁯

I do none of the things that provides me with a sense of purpose ⁯

***e) Control:***

**You feel independent and you make your own decisions**

I make all of my own decisions and can be independent ⁯

I make a lot of my own decisions and can be independent ⁯

I make some of my decisions and can be fairly independent ⁯

I make a little of my own decisions and can be a little independent ⁯

I make none of my own decisions and can’t be independent ⁯
