# Supplementary material for: Functional health state description and valuation by people aged 65 and over: a pilot study
Source: BMC Geriatr. 2018 Jan 16;18:11. doi: 10.1186/s12877-018-0711-9 (PMC5769375; doi:10.1186/s12877-018-0711-9)
Supplement: Supplementary file 4 — EQ5D + C questionnaire. EQ5D + C questionnaire utilized in the study. (DOCX 12 kb) [file 12877_2018_711_MOESM4_ESM.docx]

**Appendix D:**

By placing a tick in one box in each group below, please indicate which statements best describe your own health state today.

**Mobility**

I have no problems in walking about 􀂉

I have some problems in walking about 􀂉

I am confined to bed 􀂉

**Self-Care**

I have no problems with self-care 􀂉

I have some problems washing or dressing myself 􀂉

I am unable to wash or dress myself 􀂉

**Usual Activities** (*e.g. work, study, housework, family or*

*leisure activities)*

I have no problems with performing my usual activities 􀂉

I have some problems with performing my usual activities 􀂉

I am unable to perform my usual activities 􀂉

**Pain/Discomfort**

I have no pain or discomfort 􀂉

I have moderate pain or discomfort 􀂉

I have extreme pain or discomfort 􀂉

**Anxiety/Depression**

I am not anxious or depressed 􀂉

I am moderately anxious or depressed 􀂉

I am extremely anxious or depressed 􀂉

**Cognition (**memory, concentration, coherence, IQ)**:**

I have no problems with cognitive functioning 􀂉

I have some problems with cognitive functioning 􀂉

I have extreme problems with cognitive functioning 􀂉
