# Supplementary material for: Functional health state description and valuation by people aged 65 and over: a pilot study
Source: BMC Geriatr. 2018 Jan 16;18:11. doi: 10.1186/s12877-018-0711-9 (PMC5769375; doi:10.1186/s12877-018-0711-9)
Supplement: Supplementary file 5 — TTO example. Example of the Time Trade Off method utilized in the study. (DOCX 11 kb) [file 12877_2018_711_MOESM5_ESM.docx]

**Appendix E:**

*TTO example*

Health state **111111**

**111111**

No problems walking about

No problems washing and dressing myself

No problems with performing usual activities

No pain or discomfort

No problems with anxiety or depression

No problems with cognitive functioning

Target health state **212111**

**212111**

Some problems walking about

No problems washing and dressing myself

Some problems with performing usual activities

No pain or discomfort

No problems with anxiety or depression

No problems with cognitive functioning

How much time in state **111111** is equivalent to 10 years spent in the **target state?**

Immediate death ________

1-12 months ________

1-5 years ________

6-10 years ________

11-15 years ________

16-20 years ________

more than 20 years ________
