# Supplementary material for: Functional health state description and valuation by people aged 65 and over: a pilot study
Source: BMC Geriatr. 2018 Jan 16;18:11. doi: 10.1186/s12877-018-0711-9 (PMC5769375; doi:10.1186/s12877-018-0711-9)
Supplement: Supplementary file 6 — VAS example. Description of data: Example of the Visual Analogue Scale utilized in the study. (DOCX 13 kb) [file 12877_2018_711_MOESM6_ESM.docx]

**Appendix F:**

*VAS example*

**112112**

No problems walking about

No problems washing and dressing myself

Some problems with performing usual activities

No pain or discomfort

No problems with anxiety or depression

Some problems with cognitive functioning

**Best imaginable health state**

**-100-**

-

-

-

**-**

**--**

-

-

-

-

**-90-**

-

-

-

**-**

**--**

-

-

-

-

**-80-**

-

-

-

**-**

**--**

-

-

-

-

**-70-**

-

-

-

**-**

**--**

-

-

-

-

**-60-**

-

-

-

**-**

**--**

-

-

-

-

**-50-**

-

-

-

**-**

**--**

-

-

-

-

**-40-**

-

-

-

**-**

**--**

-

-

-

-

**-30-**

-

-

-

**-**

**--**

-

-

-

-

**-20-**

-

-

-

**-**

**--**

-

-

-

-

**-10-**

-

-

-

**--**

**-**

-

-

-

**-0-**

**Worst imaginable health state**
